# Evaluation of a multimodal pain rehabilitation programme in primary care based on clinical register data: a feasibility study

**DOI:** 10.1017/S1463423619000884

**Published:** 2020-01-14

**Authors:** Charlotte Post Sennehed, Kjerstin Stigmar, Birgitta Grahn, Marcelo Rivano Fischer, Malin Forsbrand, Anja Nyberg, Ingemar F. Petersson, Sara Holmberg

**Affiliations:** 1 Faculty of medicine, Department of Clinical Sciences Lund, Orthopedics, Lund University, Lund, Sweden; 2 Department of Research and Development, Region Kronoberg, Växjö, Sweden; 3 Department of Health Sciences, Medical Faculty, Lund University, Lund, Sweden; 4 R&D Department, Skåne University Hospital, Lund, Sweden; 5 Faculty of Medicine, Research Group Rehabilitation Medicine, Health Sciences Center, Lund University, Lund, Sweden; 6 Blekinge Center of Competence, Region Blekinge, Karlskrona, Sweden; 7 Department of Healthcare Governance, Region Skåne, Malmö, Sweden; 8 Division of Occupational and Environmental Medicine, Institute of Laboratory Medicine, Lund University, Lund, Sweden

**Keywords:** back pain, feasibility, musculoskeletal pain, multimodal rehabilitation, primary care, prospective study, sick leave

## Abstract

**Aim:**

Investigate the feasibility of identifying a well-defined treatment group and a comparable reference group in clinical register data.

**Background:**

There is insufficient knowledge on how to avert neck/back pain from turning chronic or to impair work ability. The Swedish Government implemented a national multimodal rehabilitation (MMR) programme in primary care intending to promote work ability, reduce sick leave and increase return to work. Since randomised control trial data for effect is lacking, it is important to evaluate existing observational data from clinical settings.

**Methods:**

We identified all unique patients with musculoskeletal pain (MSP) diagnoses undergoing the MMR programme in primary care in the Skåne Health care Register (*n* = 2140) during 2010–2011. A reference cohort in primary care (*n* = 56 300) with similar MSP diagnoses, same ages and the same level of sick leave before baseline was identified for the same period. The reference cohort received ordinary care and treatment in primary care. The final study group consisted of 603 eligible MMR patients and 2874 eligible reference patients. Socio-economic and health-related baseline data including sick leave one year before up to two years after baseline were compared between groups.

**Findings:**

There were significant socio-economic and health differences at baseline between the MMR and the reference patients, with the MMR group having lower income, higher morbidity and more sick leave days. Sick leave days per year decreased significantly in the MMR group (118–102 days, *P* < 0.001) and in the reference group (50–42 days, *P* < 0.001) from one year before baseline to two years after.

**Conclusions:**

It was not feasible to identify a comparable reference group based on clinical register data. Despite an ambitious attempt to limit selection bias, significant baseline differences in socio-economic and health were present. In absence of randomised trials, effects of MMR cannot be sufficiently evaluated in primary care.

## Introduction

Musculoskeletal pain (MSP), mainly back and neck pain, is one of the major causes of decreased work ability in Western countries (Gerdle *et al*., 2008; Bevan *et al*. 2009; Vos *et al*., 2012). Patients with MSP constitute a large group seeking care in primary care (Jordan *et al*., 2010; Kinge *et al*., 2015), rendering high costs both for the individual and society (Alexanderson and Norlund, 2004; Gustavsson *et al*., 2012). Early identification using prognostic screening has in recent studies shown positive results both in terms of patients’ improved health and cost-effectiveness (Hill *et al*., 2011; Forsbrand *et al*., 2017). There is various treatment options aimed at preventing acute/subacute back pain to deteriorate into chronic problems with decreased work ability, but there is insufficient evidence for these treatments (Swedish Agency for Health Technology Assessment and Assessment of Social Services; Waddell, 1987; Waddell and Burton, 2005).

There is moderate evidence for multidisciplinary rehabilitation compared to usual care (Kamper *et al*., 2015). Multidisciplinary rehabilitation has shown positive effect on function (Guzman *et al*., 2001) and there is evidence to support the use of advice to remain active (Liddle *et al*., 2007). When multidisciplinary rehabilitation has been combined with workplace interventions, it has been found to be effective (Williams *et al*., 2007; Kuoppala *et al*., 2008). In Scandinavian settings, it is reported that multimodal rehabilitation (MMR) increases return-to-work (RTW) rate (Norlund *et al*., 2009; Bakshi *et al*., 2011; Busch *et al*., 2011a). Cost-effectiveness of multidisciplinary rehabilitation programmes has been found satisfactory (Jensen *et al*., 2009; Lin *et al*., 2011). The evidence is based on mixed populations from both specialised and primary care settings.

Cross-country differences in national regulations on economical compensation for back pain seem to contribute to differences in RTW rate, while health, medical interventions and patients’ characteristic are reported as less important (Anema *et al*., 2009). European countries have varying levels of workplace health and safety provisions, which may influence work environment and the impact of adverse working conditions on health (Bambra *et al*., 2014). In Sweden, employers by law have an extensive responsibility for work environment (The Work Environment Act) in order to provide employees with safe work conditions. Employers also compensate for the first 14 days of sick leave.

In order to promote work ability, the Swedish Government implemented a national programme of evidence-based rehabilitation in primary care in 2009 (Regions). The intention with the national rehabilitation programme was to promote work ability, reduce sick leave and the risk of sick leave and increase RTW for patients with long-lasting (>3 months) MSP diagnosis and mild-to-moderate mental disorders in working age. Patients with MSP diagnoses, with ongoing pain-related sick leave or at risk of sick leave were to be offered MMR (Regions). The implementation posted many challenges concerning remittance and competence skill development (Brämberg *et al*., 2015). There was an intention to evaluate goal achievement, but there were no specific guidelines for the design of controlled follow-up. In the programme, there were no instructions to include workplace interventions, for example, contact with the employer, something that in studies have been found to have effect on RTW (Williams *et al*., 2007; Kuoppala *et al*., 2008). Economic incentives for primary care centres to provide MMR rehabilitation were introduced. The Swedish Government initially compensated the county councils with 45 000 SEK (4791 EUR) per treated patient. There were some prerequisites, for example, that MMR should involve several professions, such as physicians, physiotherapists, psychologists and occupational therapists, and that MMR should primarily be provided as group treatment. MMR should include medical treatment, physiotherapy, cognitive behavioural therapy and patient education. Furthermore, MMR according to the programme required full- or part-time attendance over four to eight weeks.

The difficulties encountered in implementation and the problems faced when evaluating MMR might partly be explained by the fact that MMR was introduced in primary care after being developed within specialised, tertiary care where previous positive results had been reported (Jensen *et al*., 2009; Norlund *et al*., 2009; Busch *et al*., 2011a; Lin *et al*., 2011). MMR within the national programme of rehabilitation has been evaluated with special focus on action, implementation and development. The implementation pace was in general slow, partly due to uncertainty regarding new treatment modalities, organisational ambiguities and uncertainties concerning patient assortment as well as lack of knowledge how to promote work ability and RTW (Brämberg *et al*., 2015). However, other evaluations found that the personnel attitudes in primary care towards extended operations were positive in focusing psychosocial interventions for patients with MSP (Bakshi, 2011; Busch *et al.*, 2011b). Much attention in the evaluations of MMR has been on patient’s outcomes (Stigmar *et al*., 2013) and on process (Norlund *et al*., 2009; Busch *et al*., 2011b). There were no published randomised controlled trials (RCTs) evaluating the effect of MMR within primary care before the implementation of the national rehabilitation programme.

There have been attempts to design follow-up studies with reference groups retrieved from register data. The Swedish Social Insurance Inspectorate (ISF) analysed MMR outcomes in comparison with treatment as usual (ISF). They applied a matching approach using broad register data to identify reference pain patients not given MMR treatment. The ISF concluded that MMR was expensive and not cost-effective, mainly due to increased sick leave during and after MMR, compared to the treated as usual group. Busch *et al*. (2017) studied the effect of MMR on sick leave by applying a matching approach using broad register data to identify reference pain patients (Busch *et al*., 2017). They found that MMR did not reduce sick leave compared to treatment as usual. However, this study included MMR both within specialised rehabilitation clinics and in primary care. In addition, a large proportion of the included patients were on disability pension at baseline and hence not representing the intended target group for MMR. An assumption behind a retrospective matching design is that there is a random distribution of MSP patients receiving MMR or not. However, referral to MMR has been found to be associated with caregiver and community-related factors (Sennehed *et al*., 2017). In summary, there is a lack of controlled trials and evaluations of MMR in primary care, especially for patients on shorter sick leave.

The aim of this study was to investigate the feasibility of identifying a well-defined treatment group and a comparable reference group in clinical register data for potential effect evaluation of MMR for MSP patients in primary care.

## Materials and methods

### Design

We performed a register-based longitudinal observational cohort study in primary care, with two-year follow-up of patients given MMR and a well-defined register-based reference group. This study examined the feasibility (Bowen *et al*., 2009) of identifying the two cohorts for an evaluation of the MMR programme. We used register data from the Population Register in Region Skåne, the Skåne Health Care Register (SHR), the Statistics Sweden, the Swedish Social Insurance Agency (MiDAS) and The National Board of Health and Welfare. We collected data from one year before baseline (start of treatment) to two years after baseline.

### Study population

The study population was identified in SHR during the period 1 January 2010 to 31 December 2011. All patients of working age, 20–60 years, registered as having received MMR in Region Skåne were identified in the SHR covering all publicly tax-financed health care in Region Skåne. A reference cohort was identified in the same register, including patients in the same age span that had been registered with the same MSP diagnose codes during the same period.

**Figure 1.**
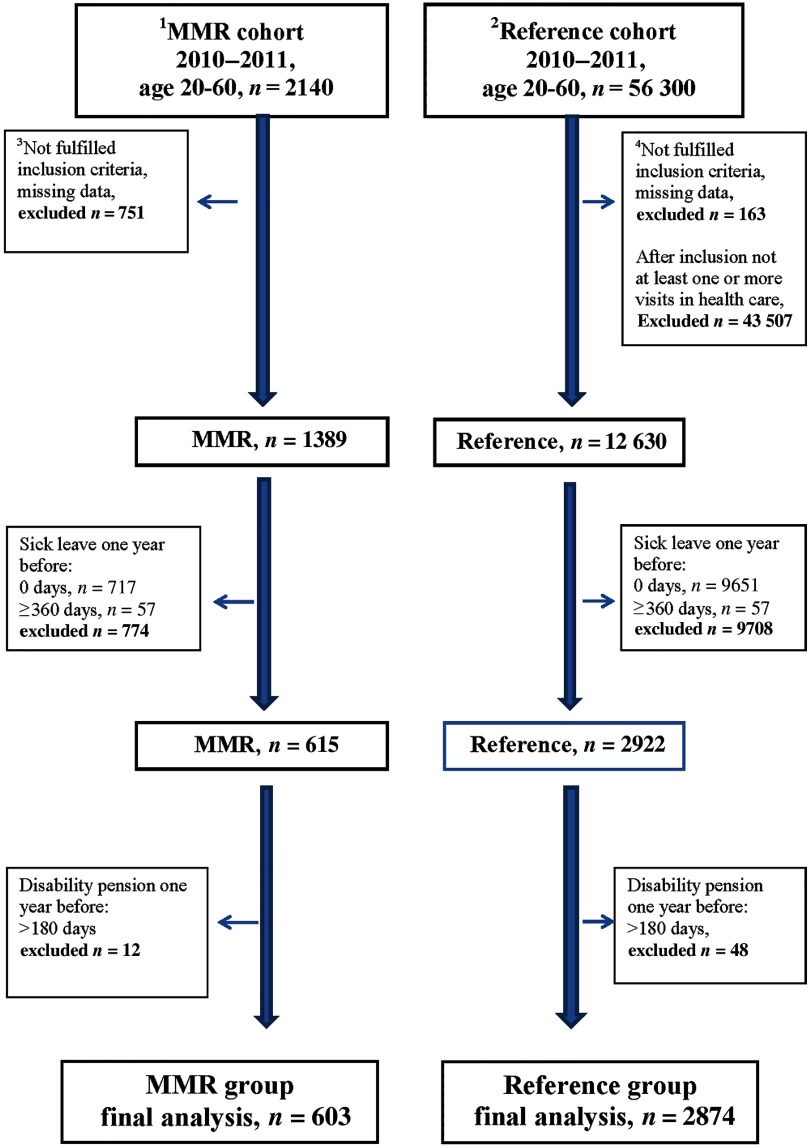
Flow chart of selection of the cohorts during 2010–2011, in age 20–60 years with MSP diagnoses Multimodal rehabilitation cohort.Reference cohort.MMR group inclusion criteria: in a register 20100101-20111231, age 20–60 years, diagnostic code according to the National Rehabilitation Program, no withdrawal, residence in Region Skåne, or deceased through 2013, no reused personal code number. At least six treatment sessions within MMR treatment period. Minimum 6 and maximum 26 weeks as MMR treatment period. At least six treatment sessions over a period of maximum six weeks. Over this six-week period at least one treatment session during four weeks in the six-week period. Either no visit or at most one visit to the rehabilitation medicine centre with a registered pain diagnosis (in accordance with the National Rehabilitation Program) over the years 2008–2012.Reference group inclusion criteria: age 20–60 years, in Region Skåne medical record with no withdrawal, any registered diagnostic code within the definition from the National Rehabilitation Program as main diagnose within Hälsovalet during 20100101-20111231, code registered at any office visit, within defined care area (primary care, psychiatric or somatic care), outpatient care, diagnose registered by physician, physiotherapist, psychologist or team, residency in Region Skåne or deceased, no reused personal code number. Either no visit or at most one visit to the rehabilitation medicine centre with a registered pain diagnosis (in accordance with the National Rehabilitation Program) over the years 2008–2012. After inclusion, at least one or more office visits within three months (92 days) that fulfil the same criteria regarding type and setting for MMR diagnose.

We identified a cohort of 2140 MMR patients, with MSP diagnoses. Approximately one-third of the patients had incomplete treatment sessions of MMR or insufficient time for follow-up and were therefore excluded (Figure 1). We identified a reference cohort of 56 300 patients with the same MSP diagnoses. In order to limit the reference cohort to patients that probably had a need for more extensive rehabilitation, we set an inclusion criterion for patients in the reference cohort, namely that these patients should have at least one or more additional consultations in health care within three months due to the same diagnosis. Three-quarters of patients in the original reference cohort did not have an additional consultation due to the same diagnose within three months and were hence excluded. In a next step, patients with no sick leave, sick leave ≥360 days and those already granted a disability pension more than 180 days the year before baseline were excluded in both the MMR cohort and the reference cohort. Non-eligible patients were excluded before baseline, and no further exclusions were made during analysis (Figure 1). The final study group consisted of 603 MMR patients and 2874 reference patients, and all study participants had been on registered sick leave at least one day up to 359 days the year before baseline (Figure 1).

### Studied variables

Age, sex, diagnoses and health care consultations were available from the SHR. Drug use measured as defined daily dose (DDD) was received from the National Board of Health and Welfare. Education, profession, net income and employment status were obtained from the Statistics Sweden (SCB). Sick leave data were received from MiDAS.

The diagnoses were registered according to the Swedish translation of International Classification of Diseases and Related Health Problems (ICD) 10 system, chapter XIII (**WHO**). Diagnoses were categorised into four groups; myalgia and pain (M245, M542, M791, M799P, R52, M840P) neck/shoulder pain (M50, M530-531, M750-755, M759P, S134, T918A), back pain (M51, M533, M543-546, M549P, M431) and rheumatism (M790). Number of contacts/year with physicians in health care was reported as mean values. Drug uses as DDD with regard to pain, sleep and depression medications were reported as mean values. Educational level was categorised as low (<10 years education), medium (10–12 years education) and high (>12 years education). Profession area was categorised into four groups; white collar (managers, senior officials, where specific education is required), pink collar (care, office, service, sale), blue collar (industry, craftsmen, agriculture, forestry, fishing) and low level (cleaning, assistants, where specific training is not required) (Yang *et al*., 2016). Net income/year (income after taxes) was reported as mean values.

Sick leave was assessed as total number of sick leave days (≥15 sick leave days 0–24 months after baseline). Sick leave days were delivered as whole days and not possible to transform to partial days. In Sweden, the first 14 days of sick leave are paid by the employer and were hence not possible to retrieve from the registry. A sick leave ≥15 day is economically compensated by the Swedish Social Insurance Agency and in the registry.

### Statistical analyses

Baseline data were compared with one- and two-year follow-up data within the MMR group and the reference group, respectively. For the descriptive statistics, we used Fisher’s exact test and Chi-square test for proportions and t-test for continuous variables. The distribution of the variables was not normally distributed, especially the number of sick leave days at baseline and one and two years after baseline follow-up. Based on this, we used non-parametric statistics to compare baseline data and one- and two-year follow-up data, the Wilcoxon’s signed rank test and related-samples Friedman’s two-way analysis of variance by ranks. SPSS for Windows version 24 was used for all statistical analyses. *P*-values <0.05 were considered significant.

## Findings

There were major differences between the MMR and the reference group in socio-economic and health measures at baseline (Table 1). The MMR patients were to a greater extent female compared to the reference group (*P* < 0.001). Diagnoses such as myalgia and pain were more prevalent in the MMR group (37% versus 23%, *P* < 0.001), and the MMR patients had more frequent contacts with physicians than the reference group (mean 16 contacts versus 9 contacts, *P* < 0.001). Drug use targeting pain, sleep and depression was higher in the MMR group than in the reference group. Also, the number of sick leave days during the year prior to baseline was higher in the MMR group than in the reference group (mean 118 days versus 50 days, *P* < 0.001) (Table 1). In summary, the differences in baseline characteristics between the MMR group and the reference group were significant for all studied variables except for age and education.

**Table 1.** Baseline characteristics of participants in the MMR group and in the reference group

	MMR *n* = 603				Reference *n* = 2874				
	*n*	%	Mean	SD	*n*	%	Mean	SD	*P*
Age	603		44.8	8.9	2874		44.5	9.9	0.423^a^
Woman	486	81			1199	42			<0.001^b^
Man	117	19			1675	58			
Education									0.159^c^
Very low	27	5			107	4			
Low	73	12			424	15			
Medium	352	58			1701	29			
High	151	25			634	22			
Missing	0				8	0.3			
Profession areas									<0.001^c^
White collar (managers/senior officials/professions where specific education requires)	138	23			562	20			
Pink collar (care/office/service/sale)	268	44			1046	36			
Blue collar (industry/craftsment/agriculture/forestry/fishing)	96	16			792	27			
Low level (cleaning/assistants/professions where specific training not requires)	82	14			344	12			
Missing	19	3			130	5			
Employment status (number in employment)^d^	534	89			2733	95			<0.001^c^
Net income, Swedish crowns (kronor)^d^	603		198.362	87.976	2874		217.730	101.815	<0.001^a^
Diagnosis^d^									<0.001^c^
Myalgia and pain	221	37			675	23			
Neck/shoulder pain	119	20			717	25			
Back pain	200	33			1393	49			
Rheumatism	63	10			61	2			
Other					28	1			
Number of contacts with health care, physician, all visits^d^	603		16	11	2874		9	8	<0.001^a^
Drug use, defined daily dose^d^									
pain	603		196	281	2874		82	182	<0.001^a^
sleep	603		51	176	2874		23	126	<0.001^a^
depression	603		119	227	2874		39	149	<0.001^a^
Sick leave, days net, converted to 100 % sick leave^d^	603		118	96	2874		50	72	<0.001^a^

a *t*-test.

b Fisher’s exact test.

c Chi-square test.

d The year before baseline.

Application of the described inclusion and exclusion criteria in order to define as relevant and comparable groups as possible resulted in that the finally defined MMR group (*n* = 603) was 28% of the entire registered MMR cohort (*n* = 2140). The final reference group (*n* = 2874) constituted only 5% of the initial registered reference cohort (*n* = 56 300). The MMR group was limited due to a large number not having a complete treatment or not fulfilling the sick leave criteria. As many as 717 patients in the initial cohort had no registered sick leave the year prior to treatment. For the reference cohort, the majority did not fulfil the criteria of having more than one health care contact due to the identified MSP diagnose within three months and among those who did fulfil this criterion most did not have registered sick leave. We also tried an even stricter sick leave criterion by limiting sick leave one year before baseline to 91–180 days and excluding all patients with any disability pension the year before inclusion in order to identify a group that theoretically would be most relevant for MMR treatment with the goal of faster RTW. However, this rendered the remaining study groups too small for meaningful analysis, the MMR group 6% (*n* = 130) and the reference group 0.3% (*n* = 213) of the original cohorts, respectively.

### Follow-up MMR group

**Figure 2.**
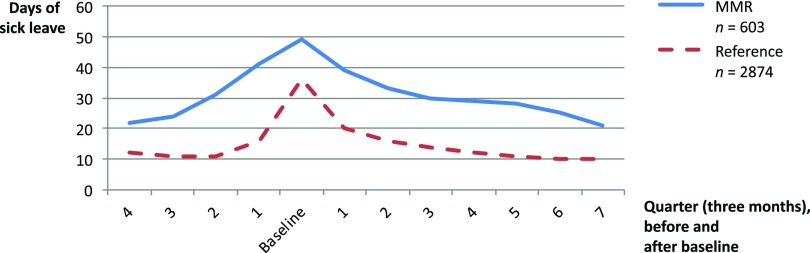
Number of sick leave days (over three months/a quarter), before and after baseline, in the MMR group and the reference group

Number of sick leave days decreased significantly in the MMR group (118 days to 102 days per year, *P* < 0.001) from one year before baseline to two years after (Table 2 and Figure 2). At two-year follow-up, 26% of the MMR patients were on sick leave lasting more than 180 days (Table 2). The proportion with no sick leave during a whole year increased from 14% one year after to 42% two years after baseline (Table 2). At baseline, 89% of the MMR patients were employed, and the proportion decreased to 78%, after two years (Table 2).

**Table 2.** Sick leave (≥15 days net) and employment status over time for the MMR group and for the reference group

Results	One year before			One year after			Two years after			
MMR group, *n* = 603	*n*	%	Mean/Md	*n*	%	Mean/Md	*n*	%	Mean/Md	*P*
Sick leave, days net	603		118/96	603		151/118	603		102/33	<0.001^a,b,c^
Sick leave, grouped										<0.001^d^
0 days				82	14		250	42		
1–90 days	289	48		171	28		113	19		
91–180 days	162	27		127	21		81	13		
181–359 days	152	25		133	22		110	18		
>359 days				88	15		48	8		
missing				2	0.3		1	0.2		
Employment staus (number in employment)	534	89		511	85		471	78		<0.001^d,e^
Reference group, *n* = 2874	*n*	%	Mean/Md	*n*	%	Mean/Md	*n*	%	Mean/Md	*P*
Sick leave, days net	2874		50/21	1656		84/42	2540		42/0	<0.001^a,b,c^
Sick leave, grouped										<0.001^d^
0 days				487	17		1611	56		
1–90 days	2373	83		1326	46		524	18		
91–180 days	288	10		424	15		189	7		
181–359 days	213	7		325	11		139	5		
>359 days				86	3		70	2		
missing				226	8		341	12		
Employment status (number in employment)	2733	95		2721	95		2461	86		0.092^d^ <0.001^e^

Related-samples Friedman’s two-way analysis of variance by ranks:

a Results one year before compared with results one years after.

b Results one year before compared with results two years after.

c Results one year after compared with results two years after.

Wilcoxon signed-ranks test:

d Results one year after compared with results two years after.

e Results one year before compared with results one years after.

### Follow-up reference group

Number of sick leave days decreased significantly in the reference group (50 days to 42 days per year, *P* < 0.001) from one year before baseline to two years after (Table 2 and Figure 2). The sick leave pattern was very similar to the MMR group although the number of sick leave days was lower over the entire period (Figure 2). At two-year follow-up, 7% were on sick leave for more than 180 days (Table 2). The proportion with no sick leave during a whole year increased from 17% one year after to 56% two years after baseline (Table 2). At baseline, 95% of the reference group were employed and the proportion was lower after two years (86%) (Table 2).

In summary, the proportion of patients on sick leave in the MMR group and in the reference group followed the same pattern with a peak the months prior to and direct following baseline. However, the MMR group had a higher proportion on sick leave over the whole study period.

## Discussion

We found it not feasible to identify two comparable groups for a fair effect evaluation due to significant selection bias. Despite an ambitious attempt to identify a comparable register-based reference group, there were significant socio-economic and health differences at baseline between the MMR group and the reference group, with the MMR group having lower income and higher morbidity. The MMR group had more sick leave days, but the pattern over time from one year before baseline and over two years of follow-up were similar for both groups.

A major strength of this study was the possibility to retrieve broad registry data with full coverage and follow-up of the patient’s over several years. In our attempt to achieve comparability between the groups, we considered it vital that patients in the MMR group and in the reference group had similar disease burden. Therefore, we used criteria to identify a reference group of patients with similar morbidity as the MMR group regarding diagnoses, health care contacts, extent of sick leave and disability pension. Despite the use of strict inclusion and exclusion criteria in order to control these conditions, the MMR group had higher morbidity compared to the reference group at baseline, represented by more complex MSP diagnoses, higher number of contacts with physicians, higher drug use and higher number of sick leave days. There were also major baseline differences between the groups in gender, profession areas and net income. Gender and age (Laisne *et al*., 2013), education and income (Streibelt and Egner, 2013), profession (Hubertsson *et al*., 2017), drug use (Rinaldo and Selander, 2016) and history of sick leave (Dekkers-Sanchez *et al*., 2008, Hubertsson *et al*., 2014) are all key variables in relation to sickness development and to successful RTW and need to be taken into account in an effect analysis. The recognised selection bias indicates lack of feasibility for the identification of a comparable reference group for effect evaluation of MMR in primary care based on observational clinical register data. It is emphasised by Craig *et al*. that the implementation of complex interventions in primary care needs to follow the rules of Medical Research Council on evaluating complex interventions (Craig *et al*., 2018).

A limitation of our study is the relatively small study population. Our ambition was to include patient cohorts from 2010 to 2012 and follow-up data up to three years after baseline. The last delivery of data was from autumn 2016, but despite this, the analysis was limited by missing sick leave data. Due to the delay in delivery of data and due to a substantial proportion missing data for the follow-up years for patients included with baseline in 2012, we restricted the analyses to baseline inclusion during 2010–2011 with follow-up over two years. The study was restricted to Region Skåne since there was a comprehensive programme for register data for all MMR treatments in the region. The sick leave data received from the Swedish Social Insurance Agency were delivered as whole days and did not include part-time sick leave days which could be considered as a limitation. It must, however, be emphasised that this was not the purpose of our study.

Using register diagnoses for inclusion of referents is problematic since diagnoses are wide entities and are affected by physicians’ assessments (Mallen *et al*., 2013). Patients registered with the same MSP diagnosis may differ in vital areas associated with work ability and sick leave. One example is the diagnosis lumbago, M545 (ICD-10), which cover everything from a first episode of mild discomfort with high work ability and no sick leave days to a period of severe pain with largely decreased work ability and a large number of sick leave days. In this study, only 28% and 5%, respectively, of the original entire cohorts fulfilled inclusion criteria. This illustrates the difficulties inherent when trying to identify relevant reference groups, based on retrospective register data for this patient population. Consequently, it questions the validity of retrospective comparisons between MSP patient groups treated with MMR and reference groups and calls for randomised study design. The national implementation of MMR in primary care can be regarded as a natural experiment, and in lack of RCTs it is worth trying to evaluate observational register data. An alternative method for evaluation might have been limiting implementation to specific areas (county councils) and kept other areas as reference in a national follow-up.

We considered a matching procedure with propensity score as an alternative method for creating a retrospective control group. However, the same difficulties in achieving enough number of reference patients can be anticipated depending on how strict matching criteria are needed in order to limit bias. From a retrospective research perspective, it is desirable that patients with similar diagnoses are referred to MMR or other treatments randomly, but from a clinical perspective we know that this is not the case. Complex clinical and practical assessments are made before decisions to refer to MMR. Referrals to MMR are also associated with caregiver and community-related factors (Sennehed *et al*., 2017). The clinical considerations made in primary care before referral to MMR are multifaceted and complex and contributes to bias by indication. Important variables such as functional level, activity level and motivation for change that should be controlled in a prospective randomisation procedure cannot be controlled in retrospect irrespective of applied method (*Grahn et al*., 2004). Finally, another option for evaluation can be to let each individual be its own control over time. This may enhance understanding of treatment mechanisms and individual effects and allow comparison between treatment subgroups. Such a design may analyse the effect of MMR in those who received it, following each person and processes (Rivano Fischer *et al*., 2019). However, without reference group, treatment effects cannot be fully assessed.

The number of sick leave days during the year before baseline varied significantly, and the MMR group was to a greater extent on sick leave in comparison with the reference group. Still, the proportion of patients with no sick leave days increased successively in both groups two years after baseline, an indicator that the treatment effect in the MMR group was similar and comparable to the reference group. The development of sick leave over time for the MMR patients if they had not received the MMR treatment is unknown. Sick leave is not a major problem for most patients with MSP consulting primary care and it is well known that about 80–90% of those with back pain recover spontaneously in about six weeks. Many do not need more comprehensive rehabilitation efforts within primary care to maintain their work ability (Waddell, 1987; Waddell and Burton, 2005). It is, therefore, important to allocate patients to the best possible treatment at an optimal time and in the case of RTW to take the number of sick leave days into account when planning treatment (Shiri *et al*., 2013; Stigmar *et al*., 2013). In this study, 25% in the MMR group and 7% in the reference group were on sick leave for more than 180 days one year before baseline. This indicates that the MMR group needed rehabilitation to a greater extent compared to the reference group and is considered a major selection bias remaining despite efforts to create comparable study groups. The sick leave criterion 1–359 of sick leave the year prior to baseline reduced the study population in both groups considerably. Only a small proportion of the study population had a granted disability pension, at most 180 days, the year before inclusion.

The MMR group differed significantly at baseline from the reference group regarding gender, profession and morbidity, well-known factors that may affect RTW and might partly explain some of the obstacles for a successful treatment (Nyberg *et al*., 2014; Kvam and Eide, 2015; Cancelliere *et al*., 2016). Myalgia and pain diagnoses were overrepresented in the MMR group and these unspecified diagnoses are well known as risk factors for failed RTW (Cancelliere *et al*., 2016; Rinaldo and Selander, 2016). Previous studies confirm the significance of important factors for RTW, such as workplace physical demands (Steenstra *et al*., 2016), social class and educational level (*Ropponen et al*., 2011), psychosocial factors (Laisne *et al*., 2013) and income before treatment (Streibelt and Egner, 2013). Furthermore, a recent study indicates that socio-economic status may influence referring to MMR (Sennehed *et al*., 2017). In the initial cohorts (MMR *n* = 2140 and reference *n* = 56 300), the proportion of patients with disability pension one year before baseline were 22% in the MMR cohort and 10% in the reference cohort. This is well in line with results presented by Busch *et al*. (2017) where a large proportion of the MMR patients showed generally worse status and a large proportion of included MMR patients were on disability pension. There was no information about the treated as usual group’s morbidity or need for rehabilitation (Busch *et al*., 2017). In our study, from the initial reference cohort, a large proportion of patients were excluded since they did not have a second health care consultation within three months due to the same MSP diagnosis. This exclusion criterion was set as an indicator of the severity of the patient’s MSP problems in order to increase the probability that the reference group had a real need for rehabilitation. We restricted the age of participants to 20–60 years in order to encompass work age in Sweden.

This study illustrates the difficulties in retrospective evaluation of medical treatments and rehabilitation. To select patients for more extensive rehabilitation, better methods are needed. Prognostic screening, as the STarT Back Screening Tool, a triage model for early identification by screening patients ‘at risk’ is one example suggested in primary care enhance the decision-making process and has shown positive results both in terms of patients’ improved health and cost-effectiveness (Hill *et al*., 2011; Forsbrand *et al*., 2017). In addition, patients’ motivation is a strong predictor for treatment results, especially for this group with pronounced pain problems (Grahn *et al*., 2004) and needs to be taken into consideration in study design. It is important to realise that rehabilitation being conducted only within the framework of health care services cannot create new suitable employments or customise employment for patients with limited work ability in their current work due to health problems. Workplace interventions, for example, contact between health care professionals and employers have been found to have effect on RTW (Williams *et al*., 2007; Kuoppala *et al*., 2008; Sennehed *et al*., 2018). More initiatives and studies involving labour market, workplace, employers and rules for conversion of work and remuneration are needed.

## Conclusion

We conclude that it was not feasible to identify a comparable reference group for effect evaluation of MMR in primary care based on clinical register data. Despite an ambitious attempt to limit selection bias, significant baseline differences in socio-economic and health status were present. In absence of some form of randomised trials, treatment effects of MMR cannot be sufficiently evaluated in primary care.

## Data Availability

Data are available from the corresponding author on reasonable request.
